# A study of the effectiveness of telepsychiatry-based culturally sensitive collaborative treatment of depressed Chinese Americans

**DOI:** 10.1186/1471-244X-11-154

**Published:** 2011-09-26

**Authors:** Albert Yeung, Kate Hails, Trina Chang, Nhi-Ha Trinh, Maurizio Fava

**Affiliations:** 1 Depression and Clinical Research Program, Massachusetts General Hospital, Boston, MA 02114, USA

## Abstract

**Background:**

Chinese American patients with Major Depressive Disorder (MDD) tend to underutilize mental health services and are more likely to seek help in primary care settings than from mental health specialists. Our team has reported that Culturally Sensitive Collaborative Treatment (CSCT) is effective in improving recognition and treatment engagement of depressed Chinese Americans in primary care. The current study builds on this prior research by incorporating telemedicine technology into the CSCT model.

**Methods/Design:**

We propose a randomized controlled trial to evaluate the acceptability and effectiveness of a telepsychiatry-based culturally sensitive collaborative treatment (T-CSCT) intervention targeted toward Chinese Americans. Patients meeting the study's eligibility criteria will receive either treatment as usual or the intervention under investigation. The six-month intervention involves: 1) an initial psychiatric interview using a culturally sensitive protocol via videoconference; 2) eight scheduled phone visits with a care manager assigned to the patient, who will monitor the patient's progress, as well as medication side effects and dosage if applicable; and 3) collaboration between the patient's PCP, psychiatrist, and care manager. Outcome measures include depressive symptom severity as well as patient and PCP satisfaction with the telepsychiatry-based care management service.

**Discussion:**

The study investigates the T-CSCT model, which we believe will increase the feasibility and practicality of the CSCT model by adopting telemedicine technology. We anticipate that this model will expand access to culturally competent psychiatrists fluent in patients' native languages to improve treatment of depressed minority patients in primary care settings.

**Trial Registration:**

NCT00854542

## Background

Chinese Americans with depression tend to underutilize specialty mental health services and seek help in primary care settings instead [1]. In an earlier study, [2] our team found that Culturally Sensitive Collaborative Treatment (CSCT) is effective in improving recognition and treatment engagement of depressed Chinese Americans in primary care. This research study proposes a randomized controlled trial to assess the acceptability and effectiveness of telepsychiatry-based CSCT (T-CSCT), which involves a culturally sensitive psychiatric consultation via videoconferencing and telephone-based care management over the course of six months. The primary goal of this study is to improve recognition, treatment engagement, and outcomes of Chinese American patients with MDD in primary care.

### Depression in Chinese Americans

Recognizing and treating symptoms of depression in ethnic minorities can be particularly challenging in populations in which depression is highly stigmatized [3]. Researchers in past studies have found that the prevalence of depression in Chinese Americans in primary care settings is relatively high, with one study reporting a prevalence rate of 19.6% [4]. Unfortunately, there are many barriers to the effective psychiatric treatment of this population, including low awareness or denial of depressive symptoms among patients themselves, as well as an under-recognition or misunderstanding of the presentation of MDD symptomatology on the part of the clinicians treating these patients [5]. Chinese Americans with depression are more likely to complain about physical and somatic symptoms, even though they will endorse symptoms of MDD [6] when they are being interviewed by clinicians or when they reply to self-report questionnaires [7]. The emphasis on somatic symptoms among depressed Asian Americans makes it harder for primary care physicians (PCPs) to identify the illness, and frequently leaves their debilitating symptoms of depression unnoticed and untreated. In addition, Chinese American patients with depression can be reluctant to characterize their depressive symptoms as a psychiatric illness due to their culture's stigmatization of mental disorders, leading them to underutilize the mental health resources that might otherwise be available to them [7].

### Barriers to Effective Cross-Cultural Healthcare

One reason that Chinese Americans tend to underutilize mental healthcare services may be a lack of culturally sensitive options for mental health treatment, including treatment that can be administered in their native language [7]. Although PCPs treating Chinese Americans (or any other immigrant population) can and should be trained in depression screening and culturally sensitive treatment alternatives, such training is not universally available [8]. Depression screening in primary care is especially important in the psychiatric treatment of Chinese Americans, who tend to seek help in primary care rather than in mental health settings [7,9,10]. However, even when PCPs are successful in screening and identifying depression in their patients, many patients stop taking a medication or continue to take the medication at an inappropriate dosage if they do not receive appropriate follow-up care. They may feel uncomfortable taking the initiative to follow up with their doctors even if their symptoms fail to improve or they experience deleterious side effects [7].

These significant barriers to effective psychiatric treatment in primary care settings could be overcome by protocols including collaborative care management. Collaborative treatment, in which mental health professionals work closely with primary care doctors in delivering mental health treatment to patients, may be a valuable option for patients who are reluctant to seek treatment outside of primary care.

Since language barriers can also lead to misdiagnosis and misunderstandings between patients and their caregivers, there is increasing interest in finding potential solutions for the dearth of Chinese-speaking clinicians to serve the needs of the growing Chinese American population in the United States. The current study will evaluate the viability of one such approach, telepsychiatry-based Culturally Sensitive Collaborative Treatment, or T-CSCT.

## Preliminary Studies

Many Chinese Americans, as well as other non-English speaking immigrants to the United States, lack access to mental health professionals who are proficient in their native language. For these populations, telepsychiatry-based collaborative management could be both a convenient and beneficial option for comprehensive healthcare.

### Culturally Sensitive Collaborative Treatment (CSCT)

The research team responsible for the current study has developed a model of Culturally Sensitive Collaborative Treatment (CSCT) [2] based on the model of collaborative care developed by Katon and colleagues [11], with the goal of using CSCT to improve the treatment outcomes for Chinese Americans with depression. Katon and colleagues [11] recommend increasing the frequency of patients' visits with their healthcare providers, with visits focusing on monitoring their treatment adherence, and alternating visits between patients' PCPs and psychiatrists. Patient education about depression and advising PCPs on appropriate depression treatment are two other important aspects of this treatment protocol. The collaborative care model has been found to help increase patients' adherence to treatment as well as their satisfaction with their care [11].

The CSCT model in the current study consists of the following five components:

### 1) Education for PCPs on the recognition and treatment of depression

PCPs are educated on the available options for treatment of patients with depression and how to improve patient adherence to treatment, one of the most significant challenges to treating depression [12]. Research shows that patient outcomes significantly improve when PCPs educate patients on how antidepressants generally work and what they can expect from taking them regularly [12]. If PCPs are briefed on the importance of having these conversations with their patients and are also informed about different treatment options for depression, patient outcomes may improve.

### 2) Screening using the Chinese Bilingual Patient Health Questionnaire-9 (CB-PHQ-9)

The CB-PHQ-9, which was validated in a study by Yeung and colleagues [13], is a brief and effective screening tool for depression that can be easily administered to Chinese-speaking patients in primary care clinics. Routine depression screening using the CB-PHQ-9 can help alert PCPs to patients' depressive symptoms that they may have missed during a relatively brief face-to-face appointment.

### 3) The Engagement Interview Protocol (EIP)

The EIP is an interview guideline of how to communicate with patients about their depression and negotiate treatment in a way that is compatible with patients' cultural beliefs, as detailed in a study by Yeung and colleagues [14]. The EIP combines standard psychiatric assessment (e.g., the Structured Clinical Interview for DSM-IV (SCID)) with patients' culturally based models for explaining mental illness. The EIP aims to enhance communication of diagnoses using a framework more easily comprehensible to patients from diverse cultural backgrounds, as well as to facilitate treatment negotiation with these populations [15].

### 4) Collaboration between PCPs and mental health professionals in the management of depression

When PCPs and mental health professionals deliver patient care in a collaborative way, patients with MDD are more likely to adhere to their medication regimen and also express more satisfaction with the quality of care they receive [11]. There is no strict definition of collaborative care; it can come in a variety of forms. The goal of collaborative care is to develop a closer working relationship between a patient's PCP and psychiatrist, with a focus on increasing the communication about a patient's treatment regimen between the two professionals. Ways to incorporate collaboration include alternating visits with a psychiatrist with visits with the patient's PCP, increasing the frequency of meetings between a patient's psychiatrist and PCP, and encouraging the psychiatrist to consult verbally with the PCP and write formal consultation notes updating the PCP of the patient's progress. These and other collaborative strategies have been strongly associated with improvement of patients' symptoms [11].

### 5) Telephone-based care management to patients with depression

Care management has been found to be very effective in improving treatment adherence and general outcomes of depression; patients also tend to report higher satisfaction with their depression treatment when they are receiving collaborative care management [15]. Collaborative care management is ideal for patients whose grasp of the American healthcare system and the English language may make it difficult for them to navigate a complex treatment regimen.

Researchers found that implementing the CSCT model in a primary care setting greatly increased the number of Chinese American patients who were diagnosed with depression and subsequently engaged in treatment, with 6.5% of depressed Chinese Americans receiving psychiatric treatment before and 43% after. In addition, patients in both care management and treatment as usual groups demonstrated a good response to treatment, although no significant differences were found between the treatment outcomes of the patients in the two groups [2]. Further research is needed to clarify why patients receiving care management did not experience even better outcomes than patients in the usual care group. One possible explanation is that PCPs in this study frequently referred study patients in both groups back to psychiatrists for treatment, so patients in both groups were receiving active psychiatric treatment that was not a part of the intervention being tested in the study.

### Telepsychiatry

Past studies have indicated that videoconferencing is both a reliable and cost-effective method of administering mental health assessments and delivering patient care [16]. When compared with face-to-face diagnostic interviews, assessments administered via videoconference have been observed to be almost as reliable [17]. In terms of the effectiveness of psychiatric interventions administered via teleconference, researchers in one study found no difference between treatment outcomes or patient satisfaction when telepsychiatry was compared to face-to-face meetings [18].

Telepsychiatry is advantageous in situations where patients lack access to clinicians who would be able to treat their mental health most effectively. It would be particularly useful for patients living in isolated or rural areas with a shortage of psychiatrists or other mental health professionals, as well as in situations where a clinician fluent in the patient's native language is unavailable.

## Study Aims

The dual aims of this study are 1) to determine whether telepsychiatry-based CSCT is acceptable to both depressed Chinese American patients and their PCPs and 2) to gain insight into the model's efficacy in improving the treatment outcomes of depressed Chinese Americans in primary care clinics.

## Methods/Design

Chinese Americans who screen positive for depression in participating primary care clinics will be called by research assistants. Eligible and interested patients will then be randomized into one of two groups. Those in the T-CSCT group will receive telepsychiatry-based Culturally Sensitive Collaborative Treatment (T-CSCT) from a multidisciplinary team, while patients in the Usual Care (UC) group will continue to receive treatment as usual from their PCPs alone. The proposed sample size is 60 patients in each group. However, anticipating a dropout rate of 20%, we aim to have at least 48 patients in each group complete all study procedures through the final week of the study.

### Description of T-CSCT Intervention

The T-CSCT intervention involves two major components:

1)T-CSCT assessment: Patients in the T-CSCT group will undergo a telepsychiatry-based culturally sensitive psychiatry assessment by a bilingual psychiatrist using the Engagement Interview Protocol (EIP), described above. Patients in the UC arm will also undergo the initial telepsychiatry-based assessment with a psychiatrist, but this assessment will not utilize the EIP.

2) Care management: The goal of care management is to monitor patients' psychiatric treatment as well as to consolidate and streamline the treatment efforts of the patient's PCP and psychiatrist. Via regularly scheduled phone visits with patients, bilingual care managers will monitor the following: patients' depressive symptoms, adherence to the MDD treatment protocol that their doctor(s) recommended, adverse events (for patients taking antidepressant medications), and patients' self-management of their depression. Care managers are accessible to patients through telephone contact or videoconferencing for questions on depression and medication, and they also can provide culturally sensitive interpretations of patients' symptoms and their treatment for depression. They serve as a link between the patient, PCP, and the consulting psychiatrist.

The first care management interview is a face-to-face meeting with a bilingual care manager to establish rapport and to explain the roles of care manager and blind assessor (see below for details on the blind assessments), as well as to review the patient's course of illness and provide an explanation of MDD and how it can be treated.

Subsequent visits will take place via telephone at seven scheduled points throughout the study (see Table 1), but care managers will make additional phone calls if deemed clinically necessary and/or helpful to patients. During each phone visit, patients' depressive symptoms will be monitored using the CB-PHQ-9. When modification in treatment is needed, the care manager will send a report to patients' PCPs with recommendations from the study psychiatrist, who will provide weekly supervision to care managers. The study psychiatrist will be available for consultation via videoconference if requested by patients, care managers, or patients' PCPs. Care managers will encourage PCPs to consider the reports on the updates of patients' conditions when deciding whether to modify patients' treatment, and they will support PCPs in implementing these recommendations. Patients who do not respond to treatment by week 10 and those who have more complicated psychiatric illnesses (e.g., psychiatric comorbidities, past treatment failures) will be encouraged to have an additional psychiatric consultation via videoconferencing.

**Table 1:** Timeline for Administration of Instruments by the Care Manager

Care Manager	Screen	^#^2 wk	^#^4 wk	^#^8 wk	^#^12 wk	^#^16 wk	^#^20 wk	^#^24 wk
CB-PHQ-9	X	X	X	X	X	X	X	X
HAM-D-17	X							
CGI-S, CGI-I	X							
Q-LES-Q	X							
Adherence, PCP Treatment*	X	X	X	X	X	X	X	X
Adherence, Medication	X	X	X	X	X	X	X	X
Adverse Events*	X	X	X	X	X	X	X	X

^# ^Care Manager will provide 8 scheduled visits to subjects in the T-CSCT Group

*Only for patients receiving antidepressant treatment from their PCPs

For patients who are receiving concurrent treatment from their PCPs, care managers will assist in scheduling follow-up visits at approximately weeks 1, 3, 6, 10, 16, and 22 of the study in order for PCPs to monitor patients' treatment response, titrate medication dosages, and manage side effects of the medications.

### Assessment by Blind Interviewers

Patients in both the intervention and usual care groups will be assessed by a bilingual blinded interviewer via phone call every six weeks throughout the six-month duration of the study. Patients receiving treatment from their PCPs will be assessed on their adherence to treatment as well as medication side effects (if applicable).

### Inclusion/Exclusion Criteria

Patients will be included if they 1) are monolingual Chinese Americans, meaning that they require or prefer to be interviewed in Chinese (Cantonese or Mandarin), 2) are 18 years of age or older, 3) are competent to consent to study participation, 4) meet criteria for MDD as diagnosed by the Mini International Neuropsychiatric Interview (MINI) [19], 5) receive a score of 10 or greater on the CB-PHQ-9, and 6) are willing to participate in phone interviews for symptom monitoring, as well as for care management if they are randomized to the treatment group.

Patients will be excluded if they 1) present with serious suicidal risk, 2) have an unstable medical illness requiring imminent hospitalization, 3) have comorbid severe mental disorders (e.g., schizophrenia, substance abuse, bipolar disorder), or 4) have been treated by a psychiatrist within the past four months.

### PCP Involvement

The study will be conducted in collaboration with PCPs at the South Cove Community Health Center (hereafter South Cove), one of the largest community health centers for Chinese Americans in the Boston area. Although we expect that most patients who enroll in the study will have PCPs at South Cove, patients who do not have South Cove PCPs will still be allowed to enroll.

Both the patients who screen positive as well as their PCPs will be informed about their screening outcomes. The PCPs of enrolled patients in both groups will be provided information about the study and must assent to treating their depressed patients who are enrolled in the study in the context of T-CSCT.

At the beginning of the study, PCPs at South Cove will attend education sessions where study staff will explain study procedures, discuss depression treatment based on the Agency for Health Care Policy and Research (AHCPR) guidelines [20], and answer any questions. PCPs who work outside of South Cove will not have the training sessions. The PCPs of patients in both the intervention and usual care arms will be notified when their patients have enrolled in the study.

In contrast to the protocol for the original CSCT study [2] on which the current study's protocol is based, patients in the current study will be provided only with consultations, but not with continued treatment, from the study psychiatrists. We hope that by controlling for extraneous aspects of patients' treatment that is unrelated to the treatment they receive as part of the study, differences in the outcomes between the usual care and intervention arms will be more prominent.

PCPs will be advised to schedule follow-up visits with the study patients at weeks 1, 3, 6, 10, 16, and 22 of the study. For patients in the T-CSCT group, the care manager will remind and assist patients in setting up these appointments; patients in the control group will set up their own appointments.

### Target Health Condition

All patients in the intervention and control arms will be recruited from patients in primary care using the PHQ-9; patients with scores of 10 or greater will be considered to have screened positive for depression.

### Recruitment Strategy

Subjects will be recruited primarily through depression screening at the three primary care clinics of South Cove, and through advertisements. Advertisements for the research study will be placed in community primary care clinics as well as in high-traffic areas in the Chinese community, in local Chinese newspapers, and on the Internet. Participants will also be recruited through referrals from primary care doctors. Research staff will distribute copies of the CB-PHQ-9 to patients in primary care clinics. Patients who score at or above a 10 on this assessment will be encouraged to call a research assistant or give the form to a nurse, who will send it to a research assistant; the RA will then contact the patient directly.

### Definition of Usual Care (UC)

PCPs of the patients who are randomized to UC will be informed that their patient is participating in the study and that the patient has depression. Patients will *not *receive protocol-driven collaborative care management; however, they may seek psychotherapy and/or psychopharmacological consultation, including a telepsychiatry-based consultation from the study psychiatrist.

### Human Subjects Approval

All study procedures have been approved by the Institutional Review Board (IRB) of the Partners HealthCare System.

## Analytic Plan

### Initial Screen

The CB-PHQ-9 will be used as the initial screening tool for patients interested in participating in the study. The CB-PHQ-9 has demonstrated promising validity, with 81% sensitivity and 98% specificity [13]. The English version of the PHQ-9 has demonstrated a sensitivity of 88% and a specificity of 88% for identifying depression in patients [21].

### Screening/Baseline Visit

In addition to the CB-PHQ-9, other instruments administered at the screening/baseline visit will include the Hamilton Rating Scale for Depression (HAM-D 17-item), the Clinical Global Impressions-Severity (CGI-S) and Improvement (CGI-I), the Quality of Life Enjoyment and Satisfaction Questionnaire (Q-LES-Q), the 16-item, Quick Inventory of Depressive Symptomatology (QIDS-SR) and the Explanatory Model of Interview Catalogue. The MINI, and the EIP (for patients in the T-CSCT arm) [22] will be administered by the clinician via videoconferencing at the screening visit.

### Care Management Assessments

After the screening visit, the care manager will provide seven additional scheduled phone visits to patients in the T-CSCT group (see Table 1). Although care managers and patients may communicate more frequently outside of those visits as clinically indicated, the assessments will be conducted eight times (including at the initial screening visit). During each of these eight care management assessments, patients will be administered the CB-PHQ-9 (to monitor their depressive symptoms)., Patients who receive mental healthcare from their PCPs will be asked about their treatment response and adherence as well as any side effects they might be experiencing from prescribed medications.

### Blind assessments

The blind assessor will contact the patient via phone call every six weeks during the six months of the study, for a total of four times (see Table 2). The instruments administered during each blind assessment will include the HAM-D-17, CGI-S, CGI-I, and Q-LES-Q. In addition, patients receiving mental healthcare from their PCPs will also be asked about their treatment response and adherence and medication side effects. Although this information will also be collected by patients' care managers for those in the intervention group, the information gathered by the blind raters will be used for data analysis (the information gathered by care managers is used more for clinical purposes).

**Table 2:** Timeline for Blind Assessment

Blind	6 wk	12 wk	18 wk	24 wk or endpoint	
Interviewer					
HAM-D-17	X	X	X	X	
CGI-S, CGI-I (subject and interviewer rated)	X	X	X	X	
Q-LES-Q	X	X	X	X	
Adherence, PCP Treatment*	X	X	X	X	
Adherence, Medication*	X	X	X	X	
Adverse Events*	X	X	X	X	

*Only for patients receiving antidepressant treatment from their PCPs

### Analysis

For all hypotheses tested, an "intent to treat" analysis examining all patients randomized to the trial will be performed to preserve the effect of randomization. Random effects linear regressions (for continuous outcomes measured repeatedly) and random effects logistic regressions (for binary outcomes measured repeatedly) will be performed to detect associations between interventions and outcomes, controlling for relevant covariates. Random effects models for repeated measures will be used, since multiple assessments will take place throughout the study. Binary outcomes that are not measured repeatedly, like treatment initiation or trial completion, will be analyzed using multivariate logistic regressions. Relevant covariates that will be controlled for in all random and fixed effect regressions include the HAM-D-17 score at the baseline visit, the use of alternative treatments for depression, study site, and gender.

One of the major goals of the study is to test the null hypothesis that the proportion of responders in the T-CSCT group is equal to the proportion of responders in the usual care group. Assuming a sample size of 48 (60 enrolled per group, with 20% attrition), an alpha of 0.05, and a two-sided alternative hypothesis, the study will have power of 0.80 to detect a statistically significant difference between the proportions of responders in the two treatment arms. This computation assumes that the difference in proportions of treatment response is 0.30 (at least 70% of patients receiving T-CSCT will meet criteria for treatment response and up to 40% of patients in the control group will meet criteria for treatment response).

## Discussion

If found to be acceptable and effective, the telepsychiatry consultation paired with collaborative care management may be an effective model in treating the mental health not only of Chinese American patients, but also of an ever diversifying American population with limited access to clinicians fluent in their culture and language.

Consultation via videoconferencing may help to widen the accessibility of culturally and linguistically competent mental health practitioners. Discussing their mental health with a clinician familiar with their culture and fluent in their native language may help patients feel more comfortable disclosing stigmatized symptoms.

This intervention may significantly increase the accessibility of mental health services to immigrant populations. The collaborative care management model may help these populations navigate the healthcare system more fluidly, thereby facilitating their adjustment to life in a new country. The current study could help clarify whether care management can or cannot improve treatment outcomes in depressed Chinese Americans, which had been questioned by a prior study [2].

If successful, the T-CSCT model has the potential to become the prototype for telemedicine-based multilingual mental health resource centers across the country, providing services to other underserved minority populations and ultimately reducing disparities in mental health treatment.

## Conclusions

The current study proposes a model that seeks to improve the mental health care of Chinese Americans at several different levels. This multifaceted model involves: 1)screening patients for depression in primary care settings, 2) having PCPs deliver effective preliminary psychiatric care before a patient can be seen by a mental health practitioner, 3) interviewing patients via telepsychiatry using a culturally sensitive psychiatric interview protocol, 4) appointing a care manager to check in with patients by phone at regular intervals, and 5) constructing a collaborative group of practitioners involved with patients' mental healthcare about how best to treat the patient.

Although this model may appear complex, we anticipate that over time, the benefits of such a model will outweigh its implementation costs, just as collaborative care models for depressed English-speaking patients in primary care have proven cost-effective [23]. This study will help determine the acceptability and effectiveness of the collaborative care/telepsychiatry model as well as bring to light any potential limitations of its design.

## Competing interests

Except for Dr. Fava and Dr. Chang, whose financial competing interests are listed below, all other contributors report they have no competing interests.

Trina Chang, MD, MPH

Dr. Chang has received research funding from AstraZeneca, CeNeRx, Euthymics, Forest, GlaxoSmithKline, Johnson & Johnson, and Pfizer.

Maurizio Fava, MD

### Lifetime Research Support

Abbott Laboratories; Alkermes, Inc.; Aspect Medical Systems; AstraZeneca; BioResearch; BrainCells Inc.; Bristol-Myers Squibb; Cephalon, Inc.; CeNeRx BioPharma; Clinical Trials Solutions, LLC; Clintara, LLC; Covidien; Eli Lilly and Company; EnVivo Pharmaceuticals, Inc.; Euthymics Bioscience, Inc.; Forest Pharmaceuticals, Inc.; Ganeden Biotech, Inc.; GlaxoSmithKline; Icon Clinical Research; i3 Innovus; Johnson & Johnson Pharmaceutical Research & Development; Lichtwer Pharma GmbH; Lorex Pharmaceuticals; NARSAD; NCCAM; NIDA; NIMH; Novartis AG; Organon Pharmaceuticals; PamLab, LLC.; Pfizer Inc.; Pharmavite^® ^LLC; Photothera; Roche; RCT Logic, LLC; Sanofi-Aventis US LLC; Shire; Solvay Pharmaceuticals, Inc.; Synthelabo; Wyeth-Ayerst Laboratories

### Advisory/Consulting

financial remuneration is $0 unless otherwise noted

Abbott Laboratories; Affectis Pharmaceuticals AG; Alkermes, Inc.; Amarin Pharma Inc.; Aspect Medical Systems; AstraZeneca; Auspex Pharmaceuticals; Bayer AG; Best Practice Project Management, Inc.; BioMarin Pharmaceuticals, Inc.; Biovail Corporation; BrainCells Inc; Bristol-Myers Squibb; CeNeRx BioPharma; Cephalon, Inc.; Clinical Trials Solutions, LLC; CNS Response, Inc.; Compellis Pharmaceuticals; Cypress Pharmaceutical, Inc.; DiagnoSearch Life Sciences (P) Ltd.; Dinippon Sumitomo Pharma Co. Inc.; Dov Pharmaceuticals, Inc.; Edgemont Pharmaceuticals, Inc.; Eisai Inc.(**2010-5500**.); Eli Lilly and Company; ePharmaSolutions; EPIX Pharmaceuticals, Inc.; Euthymics Bioscience, Inc.; Fabre-Kramer Pharmaceuticals, Inc.; Forest Pharmaceuticals, Inc.; GenOmind, LLC; GlaxoSmithKline; Grunenthal GmbH; i3 Innovus; Janssen Pharmaceutica; Jazz Pharmaceuticals, Inc.; Johnson & Johnson Pharmaceutical Research & Development, LLC **(2010-$1500; 2010-$4000.)**; Knoll Pharmaceuticals Corp.; Labopharm Inc.; Lorex Pharmaceuticals; Lundbeck Inc.; MedAvante, Inc.; Merck & Co., Inc.; MSI Methylation Sciences, Inc.; Naurex, Inc.; Neuronetics, Inc.; Novartis AG; Nutrition 21; Orexigen Therapeutics, Inc.; Organon Pharmaceuticals; Otsuka Pharmaceuticals; PamLab, LLC.; Pfizer Inc.(**2011-$3500**.); PharmaStar; Pharmavite^® ^LLC.; PharmoRx Therapeutics; Precision Human Biolaboratory; Prexa Pharmaceuticals, Inc.; Puretech Ventures; PsychoGenics; Psylin Neurosciences, Inc.; Rexahn Pharmaceuticals, Inc.; Ridge Diagnostics, Inc.; Roche; RCT Logic, LLC; Sanofi-Aventis US LLC.; Sepracor Inc.; Servier Laboratories; Schering-Plough Corporation; Solvay Pharmaceuticals, Inc.; Somaxon Pharmaceuticals, Inc.; Somerset Pharmaceuticals, Inc.; Sunovion Pharmaceuticals; Synthelabo; Takeda Pharmaceutical Company Limited; Tetragenex Pharmaceuticals, Inc.; TransForm Pharmaceuticals, Inc.; Transcept Pharmaceuticals, Inc.; Vanda Pharmaceuticals, Inc.

### Speaking/Publishing

financial remuneration is $0 unless otherwise noted

Adamed, Co; Advanced Meeting Partners; American Psychiatric Association; American Society of Clinical Psychopharmacology; AstraZeneca; Belvoir Media Group for editing a newsletter (**2008-$12,000.; 2009-$12,000.; 2010-$12,000.; 2011-$3000.)**; Boehringer Ingelheim GmbH; Bristol-Myers Squibb; Cephalon, Inc.; CME Institute/Physicians Postgraduate Press, Inc. for editing supplements & CME web activity **(2008-$5500.; 2009-$8500.; 2010-$750.; $3500. 2011) **; Eli Lilly and Company; Forest Pharmaceuticals, Inc.; GlaxoSmithKline; Imedex, LLC; MGH Psychiatry Academy/Primedia; MGH Psychiatry Academy/Reed Elsevier for speaking at symposium **(2008- $13,000.; 2009-$7800.; 2010-$6535.)**;MGH Psychiatry Academy for speaking at symposium 3/26/11 (**2011-$2500.) **Novartis AG; Organon Pharmaceuticals; Pfizer Inc.; PharmaStar; United BioSource, Corp.; Wyeth-Ayerst Laboratories

### Equity Holdings

Compellis

### Royalty/patent, other income

Patent for SPCD and patent application for a combination of azapirones and bupropion in MDD, copyright royalties for the MGH CPFQ, SFI, ATRQ, DESS, and SAFER. Patent for research and licensing of SPCD with RCT Logic. Royalty from Lippincott, Williams & Wilkins for *Handbook of Psychiatric Drug Therapy *(**2010-$835**.) Royalty from Wolters Kluwer Health Inc., (**2010: $2954. for 2009; 2011-$1599. for 2010)) **World Scientific Publishing Co. Pte. Ltd. (**2011: $544. for 2010)**

## Authors' contributions

AY originated and supervised the study, completed the analyses, and led the writing of the article. KH, TC, and NT assisted with the study and the writing of the article. MF assisted with the origination and supervision of the study and the writing of the article. All authors have read and approved the final manuscript.

## Pre-publication history

The pre-publication history for this paper can be accessed here:

http://www.biomedcentral.com/1471-244X/11/154/prepub
